# The Comparative Study of the State of Conservation of Two Medieval Documents on Parchment from Different Historical Periods

**DOI:** 10.3390/ma13214766

**Published:** 2020-10-26

**Authors:** Maria Boutiuc (Haulică), Oana Florescu, Viorica Vasilache, Ion Sandu

**Affiliations:** 1 Faculty of Geography and Geology, Doctoral School of Geosciences, “Alexandru Ioan Cuza” University of Iasi, 22 Carol I Blvd., 700506 Iasi, Romania; boutiucmariahaulica@gmail.com (M.B.); of.poni@gmail.com (O.F.); 2 County Service of the National Archives Iasi, Blvd. Carol I no. 26, 700505 Iasi, Romania; 3 “Poni-Cernatescu” Museum of Iasi, 7B Kogalniceanu St., 700454 Iasi, Romania; 4 ARHEOINVEST Centrum, Institute of Interdisciplinary Research, “Alexandru Ioan Cuza” University of Iasi, 11 Carol I Blvd., 700506 Iasi, Romania; 5 Romanian Inventors Forum, Str. Sf. P. Movila 3, 700089 Iasi, Romania; 6 Academy of Romanian Scientists (AOSR), 54 Splaiul Independentei St., Sect. 5, 050094 Bucharest, Romania

**Keywords:** parchment, collagen, state of conservation, OM, SEM-EDX, ATR-FTIR

## Abstract

The paper explores the potentiality of an experimental multianalytic protocol with appropriate methodology for determining the chemical and morphostructural characteristics of two old documents on parchment support. Such a protocol can authenticate and assess the state of conservation under the influence of environmental factors during storage and archival documentation, thus advancing preventive and prophylactic measures in “treasure” deposits such as the National Archives of Romania, where these documents are kept. The work methodology consisted of three stages. The first stage consists of visual observation for identifying deteriorations and degradations, alongside the selection of representatives’ areas from where micro-samples were collected. The second stage involves Scanning Electron Microscopy coupled by Energy Dispersive X-ray Spectrometry (SEM-EDX) analysis, for highlighting the morphology and determining the elemental composition; lastly, the Fourier-transform infrared (FTIR) analysis and correlation of results establish the chemical and morphostructural changes. The use of this gradual system of analyses allowed determining the differences between these two documents in terms of the materials used for producing them, their manufacturing technologies, the writing and ornamentation, and the overall state of conservation. The results provided the first accurate picture of the chemical nature and manufacturing of the two parchment documents by determining the main characteristics of the collagen and of the finishing, writing, and decoration materials, in view of the natural aging through the oxidative and gelatinization processes of the collagen. The SEM-EDX results revealed the morphological changes of the parchment that occurred at various levels in the collagen fibrous mesh and established the state of conservation of the support, writing, and decorations.

## 1. Introduction

Parchment is obtained from untanned or slightly tanned animal skins (lamb, sheep, goat, deer, calf, oxen, camel, crocodile, seal, etc.) by removing the epidermis and hypodermis, involving various physical–chemical and mechanical treatments. The main component of the dermal tissue is collagen, which is a fibrillary protein with a framing and supporting role. The fibrous nature of the collagen is due to long sequences of amino acids (approximately 20) forming long chains, with a molecular mass of 90,000–95,000. The collagen fiber consists of three long chains, linked in α-helix [1], and each polypeptide chain has crystalline areas where the amino acids are nonpolar and amorphous areas where the amino acids are polar or polarizable. The polypeptide chains will undergo hydrolytic cleavage during the preliminary operations of removing the epidermis and hypodermis. These cleavages leave their mark on the evolution of the rate of aging, being an endogenous factor that interacts easily with exogenous ones (pollution, microorganisms, atmospheric humidity, temperature, illumination, etc.). A specific characteristic of collagen is thermal denaturation and that produced by strong acids and bases, and gelatinization in hot water; both groups of processes render parchment insoluble in water, diluted solutions of acids, bases and neutral salts, and organic solvents at normal temperatures [2,3].

Therefore, documents and other old writings on parchment, on account of their heritage value, have been at the center of various studies throughout time [4,5,6,7], aiming to identify the main components of the support, writing, and miniature materials, with regard to the evolution of the state of conservation in correlation with endogenous and exogenous factors, which affected their integrity, aesthetics, and historical message.

The use of high-resolution optical microscopy (OM), Scanning Electron Microscopy coupled by Energy Dispersive X-ray Spectrometry (SEM-EDX), and attenuated total reflection Fourier-transform infrared (ATR-FTIR) is well established for characterizing the natural degradation of old parchments [8], specifically for determining the main characteristics of the collagen and establishing their organic and inorganic composition, alongside assessments based on changes in crystallinity pertaining to natural degradation.

Similarly, there are other techniques used for assessing the state of conservation of ancient or medieval parchments [9,10], which are of particular importance for developing adequate/compatible procedures for the preventive and prophylactic preservation of valuable historical documents.

Assessing the authenticity of old documents on parchment involves a series of analysis methods that allow determining certain archaeometric characteristics or the chemometric characteristics of archaeometric value; these methods are generally used for identifying forged and counterfeit documents [11].

The present paper advances an experimental multianalytic protocol with a specific work methodology for determining the chemical and morphostructural characteristics of two old documents on parchment, in order to authenticate and establish the evolution of the state of conservation under the influence of environmental and anthropic factors during storage and archival documentation, as to warrant taking urgent preventive and prophylactic preservation measures in archival deposits.

The two medieval documents are of high historical and artistic value, were produced in different periods, and are kept by the Iasi County Service of the National Archives of Romania, where they are assigned to the “Treasure” collection. The two documents were analyzed by corroborating the OM, SEM-EDX, and ATR-FTIR techniques, in order to determine the state of conservation for establishing the evolution of structural and aesthetic–artistic integrity across time. We analyzed the chemical nature and a series of characteristics of the support, the materials used for writing, ornaments, and miniatures, which allow us to identify the evolutionary effects of deterioration and degradation.

## 2. Experimental Part

### 2.1. Description of Documents and Their State of Conservation

For this study, we selected two old parchments from the collection of medieval documents of high heritage value (classed in the treasure fund), which were created 154 years ago using very different artistic techniques and execution technologies. This study aims to obtain information regarding the nature of the component materials and their state of conservation, alongside a series of other archaeometric and morphostructural characteristics, in order to trace their integrity diachronically.

These documents were indexed with the initial S followed by the order number 1 and, respectively, 2. These documents were sorted according to their heritage value.

**Figure 1:**
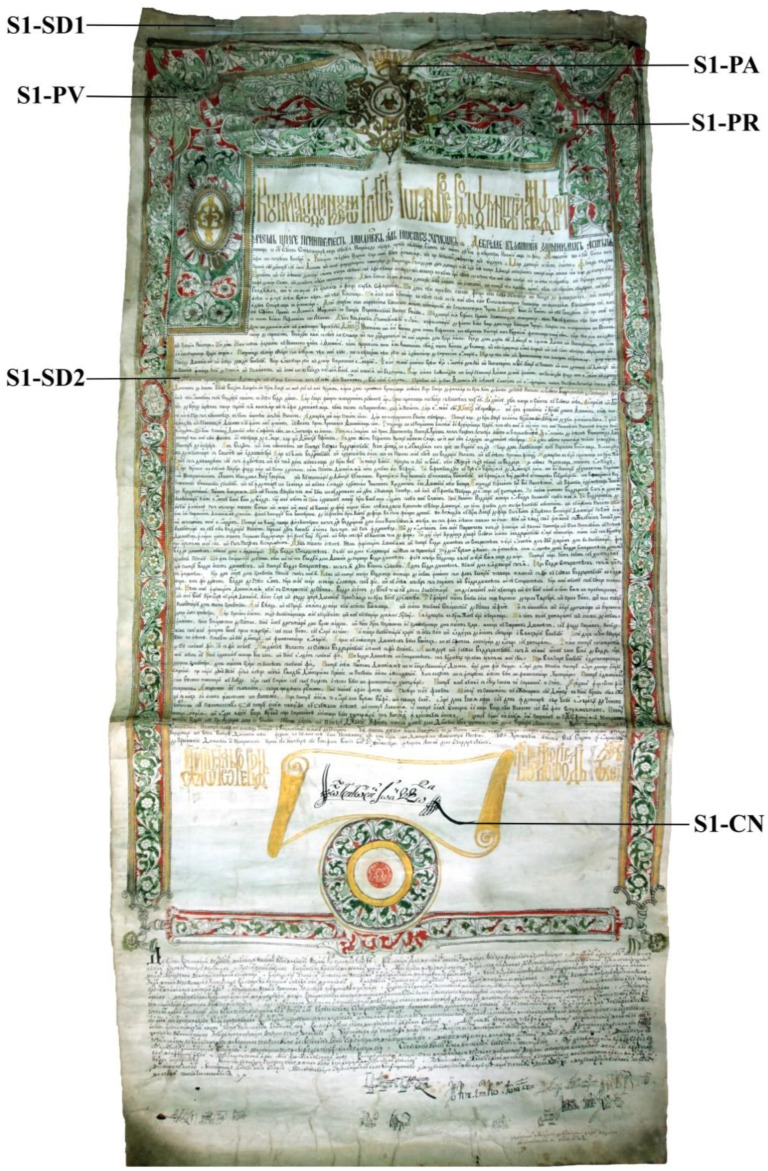
Parchment S1 with the index codes of the areas under scrutiny.

Figure 1 presents the image of document S1, which is a charter issued on 15 October, 7272/‹1763› by the princely chancellery of Moldavia, during the rule of Voivode Grigore Ioan Callimachi, which cancelled different taxes (on vineyards and winemaking).

The document is rectangular in shape and was kept rolled or folded in three; it measures 140.5 × 65.5 cm and was manufactured from three pieces of parchment by binding their overlapping edges (a 5.0 × 65.6 cm stripe at the top, a 81.5 × 65.6 cm stripe at the middle, and a 54.0 × 65.6 cm at the bottom). After hydric stabilization and cutting out, the ornaments and writing were inserted. The substrate of the document is yellow in color because the stage of tanning was skipped. Moreover, it has a silky texture, particularly the back. It is embellished with a sumptuous decoration in the form of a carefully executed frame filled with ornaments and miniatures and the coat of arms of Moldavia (an official sign acknowledged and perpetuated for centuries), an auroch head seen from the front, with a star in-between the horns. The auroch head is surrounded by Christian iconographic elements, which are surmounted by a crown. The drawing and ornaments are made in black, red, green, and golden ink. In the lower part of the document, there is the seal impression made in red ink and surrounded by decorative rosette, which in modern terms is assimilated with a stamp. The writing, in black ink, is aligned and symmetrical, while the auroch head, crown, title, symbolic invocation, and capitals are in golden ink. In addition to its documentary importance, this document is an exceptional work of calligraphic and miniature art. It is part of the richly decorated documents of Moldavia, which are much rarer than those of Wallachia. This is due to the limited attention given by the princely chancellery of Moldavia to coloristic art, even though this was a flourishing era for miniature art overall. Even though the throne of Moldavia was occupied by three princes of Wallachian origin (Radu Mihnea, Simion Movila, and Alexandru Ilias), who tried to impose the richly decorated style, calligraphic writing, and miniature decoration of documents, but without consequences in Moldavia. Such a tradition existed in this principality only in monastery workshops before the age of Stephen the Great.

When collected from the archives, the document presented degradations and denaturation in the form of cornification and crumbling. In addition, the overlapping of the components of the support and margins were fragilized, and in some areas, it was fringed, with microfibers failing to bind together. Other defects were a series of restricted areas with fouling halos and spots of tegumentary lipids contaminated with atmospheric dust, particularly the superior part of the backside of the document.

**Figure 2:**
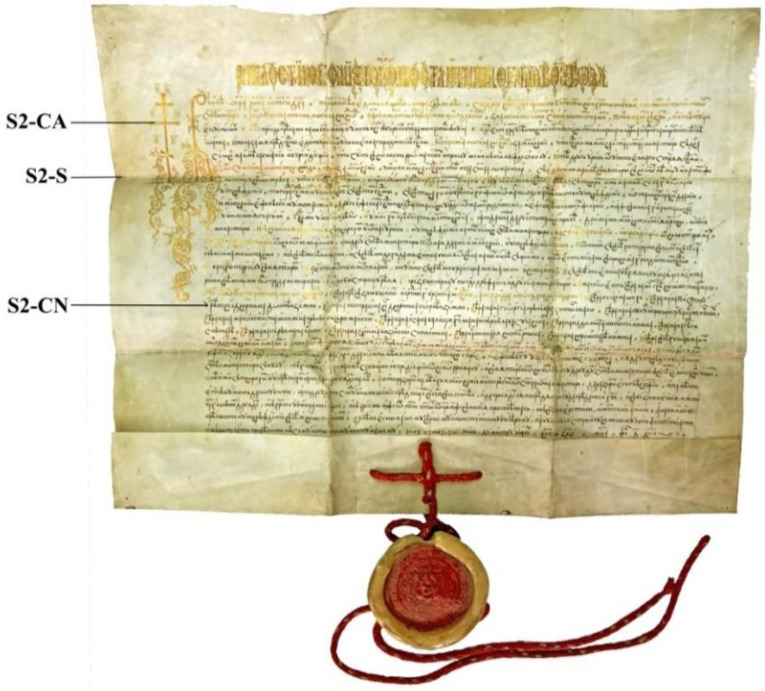
Parchment S2 with the index numbers of the analyzed areas.

The second document, indexed as S2 (Figure 2), is an original Slavonic parchment, with a hanging seal, containing the charter of Constantin Moghilă, the Prince of Moldavia, reconfirming the rights and privileges of the Secu Monastery. The document was issued in the year 7117 (1609), on 4 February, in Iasi. The manuscript is rectangular in shape, measuring 63.4 × 57.5 cm, of which 8 cm represent the width of the sleeve (the lower cuff of the document folded at the front, fitted with four rhombic perforations placed at the center, through which the string of the seal runs).

The document was kept folded in three, in a sleeve. The seal is the element that authenticates the document; it is large, round, and impressed once (on the front) on warm red wax with the coat of arms of Moldavia.

With respect to ornamentation, compared to the first document, this one is simpler, with fewer colors: black and golden. The golden ink was used for writing the title, the invocation with “the cross ahead”, the initials, the first two rows, certain expressions, and the capital letters in the body of the text. The initial and the invocation are much larger in size than the rest of the writing, of 16 and 12 rows.

This document is better preserved than the first document, but it has traces of use, with loss of material along the folding lines. All these makes the document highly fragile. The support has the collagen fibers well individualized and presents a better flexibility during manipulation.

### 2.2. Samples and Collecting Areas of Documents

A series of samples was taken from the areas marked in Figure 1 and Figure 2 and indexed according to the area, as follows:

- S1-SD, degraded collagenic support (samples S1-SD1 and S1-SD2);
- S1-CN, writing ink based on black pigment;
- S1-PR, ink used for the red-pigment miniature;
- S1-PA ink used for the golden/yellow pigment miniature;
- S1-PV ink used for the green pigment miniature;
- S2-CN, black ink;
- S2-CA, golden ink.

### 2.3. Methods and Analysis Techniques

In the first stage, the documents were the subject of direct analysis, by visual analysis, with the naked eye or with optical magnifying instruments: lens, binocular stereo lens, and stereomicroscope.

Optical microscopy was conducted using a Carl Zeiss Axio Imager A1m microscope (Berlin, Germany) at magnifications between ×50 and ×200, attached to an Axiocam camera and using specialized software.

For the SEM-EDX analysis, an SEM model VEGA II LSH produced by Tescan (Brno, Czech Republic) and coupled to an EDX detector type Quantax QX2 made by Bruker/Roentec (Berlin, Germany) has been used. The analysis of the samples was carried out at a magnification of 200…2500×, with an acceleration tension of 30 kV, and the work pressure was lower than 1 × 10^−2^ Pa. The resulting image was constituted by secondary electrons (SE) and backscattered electrons (BSE). The SEM-EDX microscopy involved the collecting and processing of the samples.

FTIR spectra were obtained using a Vertex 70 FTIR (Bruker, Berlin, Germany) equipped with accessories: ATR mode and RAMAN II. The spectra were recorded in the range of 4000–700 cm^−1^ with a resolution of 4 cm^−1^.

## 3. Results and Discussions

### 3.1. Visual Analysis

The direct visual analysis of these two documents point out that the parchment supports are in good quality, without major defects and with uniform thickness, light color, lusterless appearance, smooth, velvety surfaces, and the writing area well prepared. Both documents meet all the characteristics of a document on parchment issued by the princely chancellery of Moldavia. The deteriorations and degradations found were caused by both endogenous and exogenous factors, which left their marks on the evolution of the aging rate, respectively on the structural–functional and aesthetic–artistic integrity of the artefacts.

Document S1 (Figure 1) was analyzed by a handheld lens and stereomicroscope, and it has a series of deteriorations and degradations caused by careless handling and use, such as fine tears and frays caused by hydrolytic embrittlement and limited ungluing in the overlap area of the three constituting pieces of parchment caused by cornification processes through chemical or thermal denaturation, particularly in the top area. Regarding the writing and miniatures, document S1 presents fine erosions (thinning of the layers) and exfoliations of the writing and miniature inks caused by fluctuations of the microclimate parameters beyond the optimal values (18–22 °C for temperature, 50 lx for illumination, and 50–60% for atmospheric humidity). Pollution, illumination, and atmospheric humidity turned the originally white parchment to yellow-gray, as seen on both the front and back side of the document, particularly the top area of the document, since that was the most exposed part.

Document S2 (Figure 2) is better preserved; it is darker in color in the fold lines areas because of the dust collected on the surface. The document has small areas where the material was lost along the folding lines, particularly in the case of multiple folds, where the corners resulting from folding are highly fragile. Examination under a stereo lens highlights the substrate with well-defined fibers in terms of its morphology and entanglement system. The parchment is still sufficiently flexible for manipulation.

### 3.2. Analysis of the Texture

**Figure 3:**
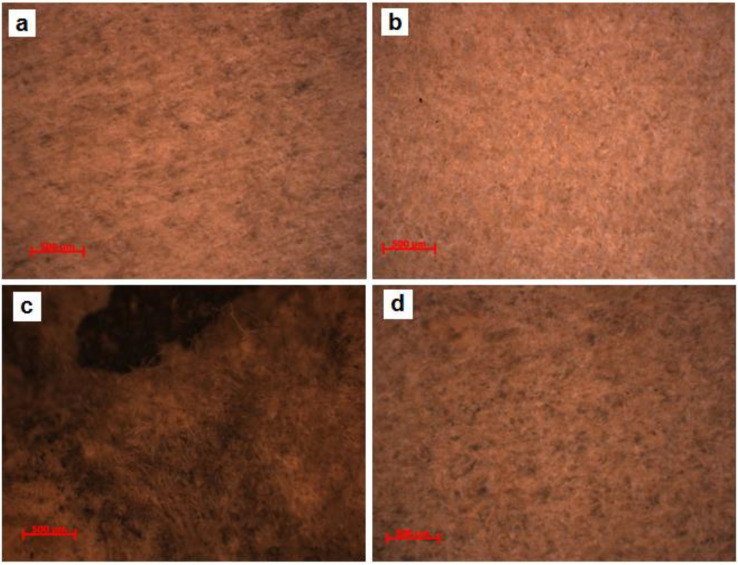
Optical microscopy (OM) images (×50) of document S1: (**a**) marginal area (S1-SD1); (**b**) between the rows of writing (S1-SD2); (**c**) the binding area of the support (S1-SD2); and (**d**) the back of the document.

**Figure 4:**
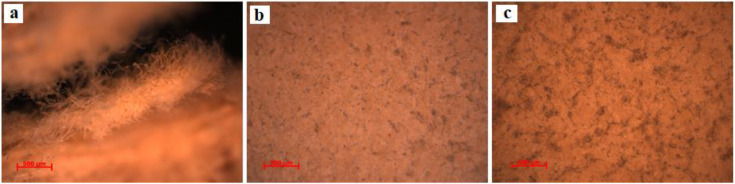
OM images (×50) of document S2: (**a**) the degraded and fringed area on the front; (**b**) well preserved area on the front; and (**c**) well preserved area on the back.

These two documents were analyzed by optical microscopy, without collecting samples, directly through reflection, in white light, dark field, using the selected areas by experimental protocol. Therefore, for document S1, we obtained microphotographs of the front of the parchment in the marginal area free of writing (S1-SD1, Figure 3a) between the written rows in the binding area (S1-SD2, Figure 3b,c) and also from the same areas of the back of the parchment (Figure 3d), while for the document S2, we analyzed from the front a degraded area (Figure 4a), one without conspicuous degradation (Figure 4b), and another one from the back of this document (Figure 4c).

Following the analysis of the granular configuration determined by the arrangement of the pilose follicles (removed by physical–chemical and mechanic processes from the top layer of the dermis), we found that both documents were obtained from veal leather, which has a denser and finer structure than mature bovine skin, and that the alveoli are smaller and the pilose follicles are distributed uniformly, in rows of one and in relatively linear lines [2,3,12].

**Figure 5:**
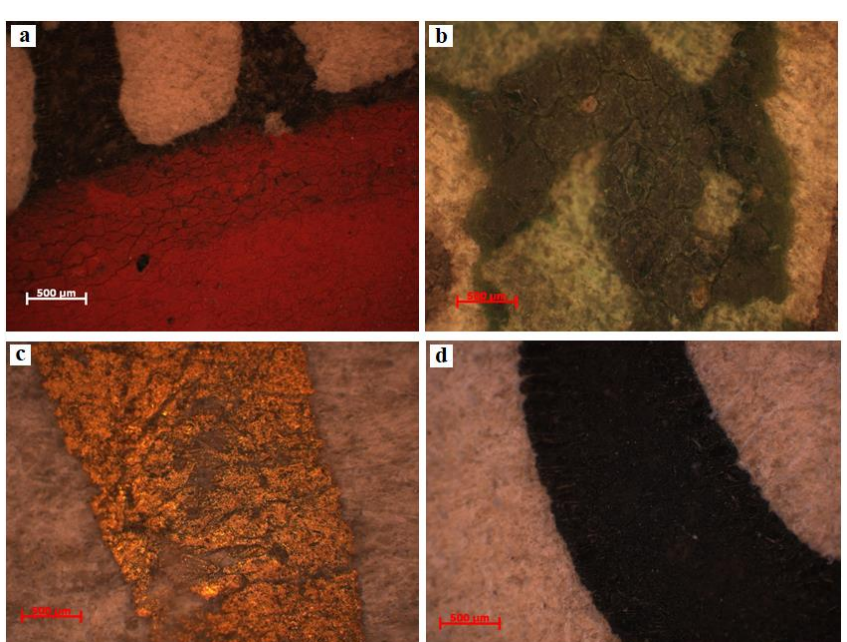
OM images (×50) for the writing and miniature inks of document S1: (**a**) S1-PR (red pigment); (**b**) S1-PV (green pigment); (**c**) S1-PA (golden pigment); and (**d**) S1-CN (black pigment).

**Figure 6:**
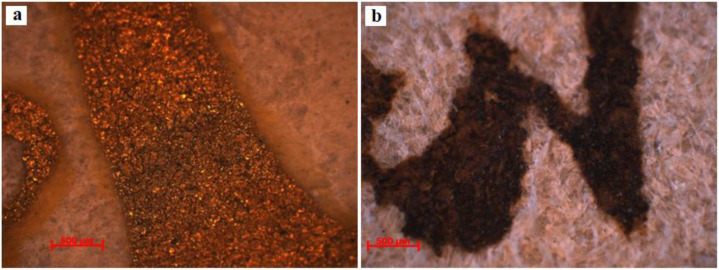
OM images (×50) for the writing and miniature inks of document S2: (**a**) S2-CA; (**b**) S2-CN.

Figure 5 and Figure 6 present the OM (×50) microphotographs for the writing and miniature inks of document S1 from the following areas: S1-PR (red pigment), S1-PV (green pigment), S1-PA (golden pigment), and S1-CN (black pigment). For S2, microphotographs were obtained for areas S2-CA (golden ink) and S2-CN (black ink).

The analysis of the images of the inks used for writing and miniatures reveals a series of aspects, as they are presented below.

The order of application of the inks on document S1 was first black ink and then red ink (Figure 5a).

Both red and green ink (Figure 5a,b) present reticular fissures (cracks) and micro-lacunae resulting from the detachment of the inks from the support. These forms of deterioration are due to the fragilization in time of the binder in the two inks, meaning that the process of hydrolytic alteration determines the destruction of the writing and miniature works. Low humidity alongside high temperature causes dehydration, which is followed by the contraction and breakup of the inks, producing age cracks.

More wear is seen in the case of the golden ink on document S1 (Figure 5c), where the detachments are more critical. In this case, besides the causes described for the red and green inks, the different hygroscopicities of the collagen support and of the two components of the golden ink (pigment and binder) are also relevant, which at fluctuant humidity produce different dilatations and contractions. On the other hand, the golden ink from document S2 (Figure 6a) has traces left by the elevated humidity of the air up to the point of saturating the fiber, causing the migration of the binder to the vicinity of its placement on the document along its contour, which causes a better attachment to the support by increasing the contact area through the resulting halo.

The microscopic analysis of the black ink from document S1 (Figure 5d), which was made from carbon pigment, showed signs of dividing and the detachment of microparticles, despite the fine macroscopic appearance. In the case of document S2, in Figure 6b, we noticed the ingress of the ink into the collagen support, which proves that the ink was ferogallic and was likewise confirmed by the SEM-EDX and ATR-FTIR analyses. However, document S2 also presents a detachment of microparticles that are darker in color, which proves that the ink was a mixture of iron gall and carbon: a recipe commonly used in the past in order to mitigate the shortcoming of illegibility of ferogallic once it is used. Since it is a solution, the iron gall ink was absorbed by the support, coloring it irreversibly, whereas carbon ink, notwithstanding its excellent stability in time, does not adhere well to the smooth surface of the parchment because the dispersed fine carbon does not enter the collagen fibers, but only affixed on the surface. It follows that the sole culprit for the degradation of the carbon ink is the binder (gum Arabic or animal glues), which is more sensitive to the action of exogenous factors.

### 3.3. The Analysis SEM-EDX

For obtaining structural details beyond those provided by optical microscopy, we employed SEM. The micro-samples analyzed by SEM-EDX were collected from the same areas as in the case of OM.

**Figure 7:**
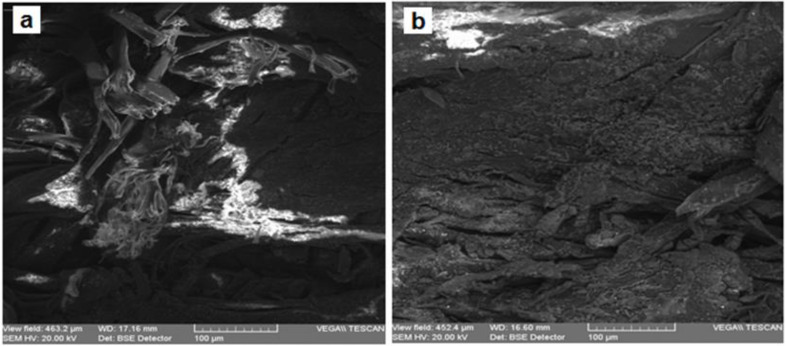
SEM microphotographs (×500, backscattered electrons (BSE)) for the support of parchment S1: (**a**) degraded support, area S1-SD1; (**b**) degraded support, area S1-SD2.

**Figure 8:**
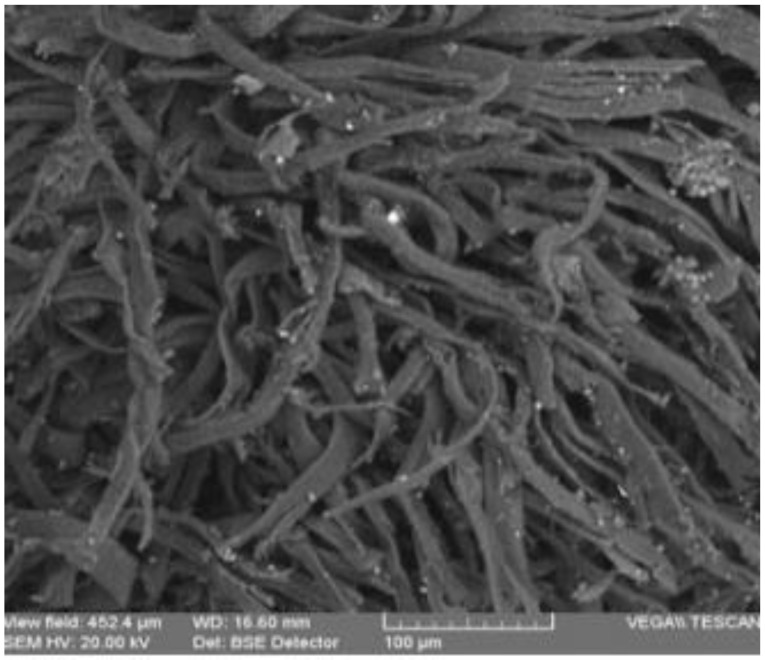
SEM microphotographs (×500, BSE) for the support of parchment S2, the degraded areas S2-S.

Figure 7 and Figure 8 present the SEM microphotographs of the two parchments for the areas most affected by destructions and alterations.

**Figure 9:**
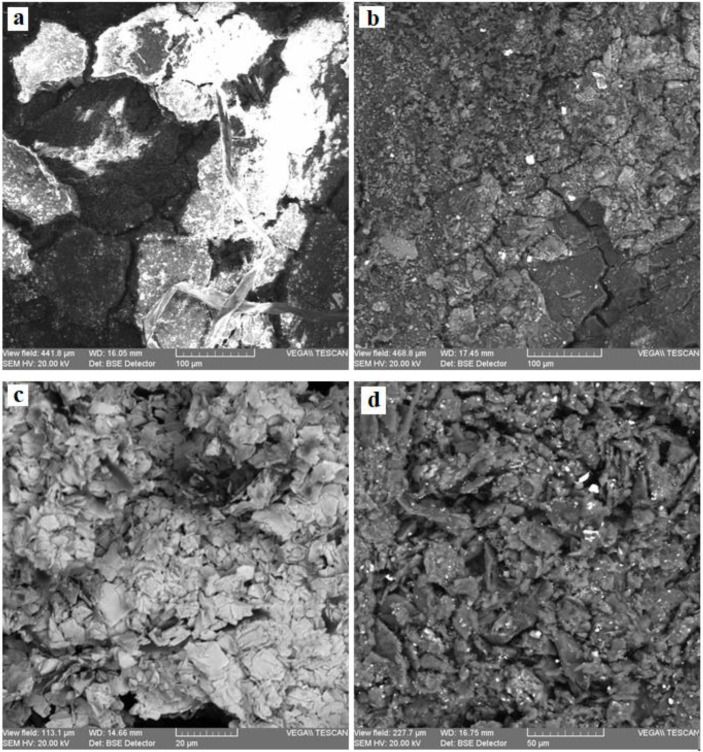
SEM microphotographs for the inks of document S1: (**a**) S1-PR (×500); (**b**) S1-PV (×500); (**c**) S1-PA (×2000); and (**d**) S1-CN (×1000).

**Figure 10:**
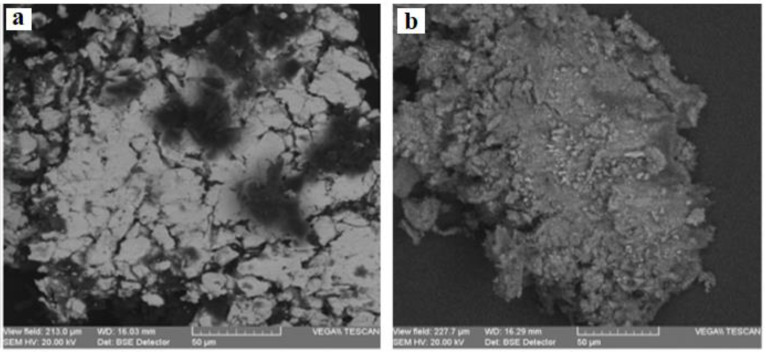
SEM microphotographs (×500, BSE) for the inks of document S2: (**a**) S2-CA; (**b**) S2-CN.

The analysis of the images from Figure 7 and Figure 8 reveals the following:

- The collagen support of document S1 is more degraded by denaturation and gelatinization, presenting fibers compacted by monolithization and with a reticulation of three-dimensional fissures and micro-crevices caused by the fracturing occurring when the document was rolled up (Figure 7a,b), with the frayed fibers clearly distinct on the surface from the monolithized ones.
- The sources of these deteriorations were initially due to alteration processes, which led to evolving effects of degradation caused by higher temperatures of the environment in which the document was kept, and by air humidity above 70%, which are optimal conditions for activating microbiological attack; subsequently, damage had also resulted from constrictions at high temperatures and environmental humidity below 40%, which led to dehydration and the loss of hygroscopic water, resulting in contractions after the elimination of the hydrogen bonds from the teloproteins of the polypeptide chains of the collagen molecules [13].
- The advanced loss of hygroscopic water down to 5% rendered the parchment supports rigid, creasy, and shriveled; in case of accidental moistening, the supports will disintegrate in thin sheets.
- The visual analysis using the handheld lens or the stereomicroscope of the various forms of shredding and fringing shows multiple amorphous areas in the collagen fibers.
- In the case of document S2, the collagen fibers are very well individualized within a 3D meshing (Figure 8), with conspicuous microparticles of calcium carbonate and inorganic salt crystallites left as residues of the preliminary processing of the skins.
- Compared to S1, document S2 is more flexible, which is noticeable during manipulation.
- The SEM images of the inks of these two documents, as presented in Figure 9 and Figure 10, confirm the results obtained by the OM analysis. Furthermore, the SEM detailing allowed a better highlighting of the cracks, exfoliations, micro-zonal lacunae, and scaly structures in the form of plates/lamellae for the golden pigment (Figure 9c and Figure 10a) and non-uniform granules, with a reticulation of fissures and micro-crevices for the black carbon-based pigment (Figure 9d and Figure 10b).

The EDX spectra provided the elemental composition of the structural components of the two documents, as presented in Table 1.

The elemental composition of the micro-samples allowed the following observations:

- The calcium in CaCO_3_ left as a residue of the manufacturing technology renders the support alkaline, making it more resistant to acids and micro-organism attack [13,14,15,16]. In the case of the support of parchment S1, the concentration of Ca is much higher (approximately five times higher) than that of S2, which shows that the technologies for obtaining the two writing supports were different. The production of S1 involved the treatment with calcium hydroxide (slaked lime), while for S2, the treatment consisted of calcium hydroxide and sodium sulfite, which expedited the removal of the epidermis and hypodermis, including their components (hairs/wool, glandes, etc.). If S1 had been stored in the same microclimate conditions as S2, the former document would have been better preserved than the latter.
- Sulfur, in the case of document S1, originates from sulfuric amino acids, having a lower value in the area most degraded (where the amorphous areas of the collagen fibers predominate), and from the sulfur found in the ink pigments diffused through migration into the support. In the case of document S2, the sulfur concentration is greater, since it originates from the treatment with sodium sulfite added in the calcium hydroxide bath and from the migration into the support of a portion of the sulfur contained by the ferogallic ink.
- The presence of aluminum and silicon in these two parchments is due to the atmospheric dust that adhered to the surface of the documents in the form of aluminosilicates and to impurities found in the calcium hydroxide used in the preparation stage of the parchment; these two elements are found in greater quantities in S1 than S2.
- Chlorine and sodium originate from the sodium chloride (salt) used for treating the hides just after skinning.
- The presence of the chemical elements potassium, magnesium, sodium, and calcium in all inks except the golden one can be explained by the use as a binder of gum Arabic, but also from the impurities found in the hydrated lime (slaked lime) used for manufacturing the parchment.
- The composition of pigments, with traces of aluminum, sodium, potassium, magnesium, and silicon suggests that they were obtained from colored earths.
- The green pigment may be natural ultramarine—3Na_2_O·3Al_2_O_3_·6SiO_2_·3Na_2_S—combined with iron yellow ochre—Fe_2_O_3_–, green apatite—Ca_5_(PO_4_)_3_(Cl, OH)—or malachite (basic copper carbonate—[2CuCO_3_Cu(OH)_2_]).
- The red pigment, with a mercury content of 8.325% and sulfur content of 1.256%, suggests that it originates from cinnabar (known in its natural form since antiquity and the artificial form since the 13th century). This pigment turns brown under the action of natural light radiation, but in our case, the document’s writing and ornamentation were protected from light by rolling and folding it.
- The golden pigment contains a large quantity of gold, specifically 70.69% in the case of parchment S1 and 52.85% in the case of S2; the golden ink of the latter document also contains traces of calcium and carbon originating from the gum Arabic and the calcium hydroxide used during the execution of the manuscript.
- The black ink used for writing and producing the ornaments of document S1 is carbon, and the one used in document S2 is a ferogallic ink combined with a small quantity of carbon.

### 3.4. Analysis by ATR–FTIR

**Figure 11:**
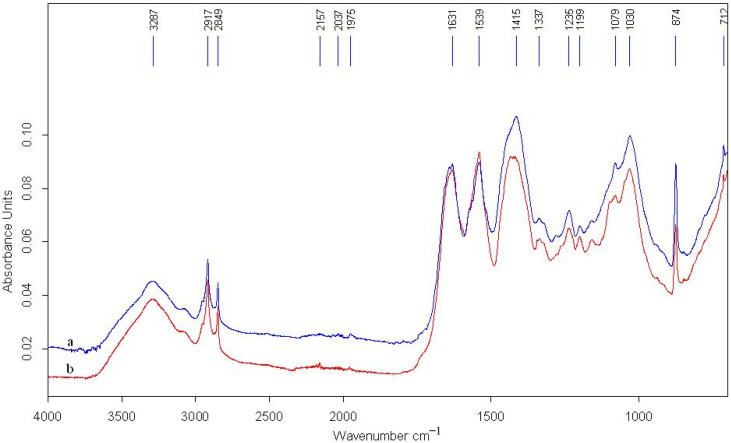
Fourier-transform infrared (FTIR) spectra on the support of the two documents: (a) S1-SD; (b) S2-S.

**Figure 12:**
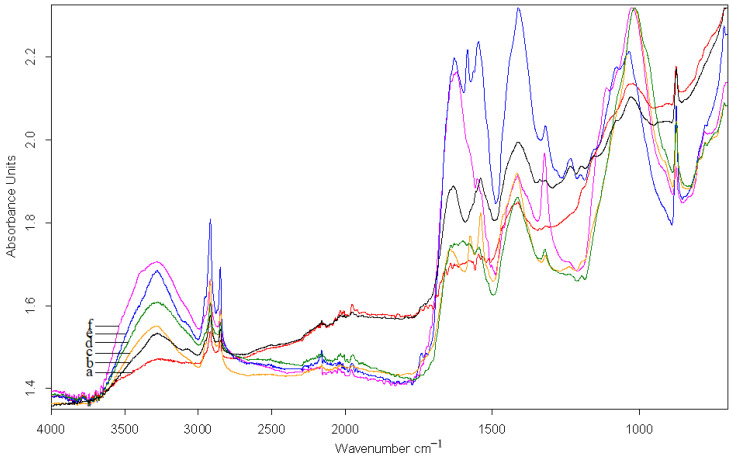
The FTIR spectra for the ink on the two documents: (a) S2-CA; (b) S1-PA; (c) S1-PR; (d) S1-CN; (e) S1-PV; and (f) S2-CN.

In order to highlight the degradations of these supports and inks (pigments and binders), the spectrum of these two supports were overlapped in Figure 11 and respectively in Figure 12. Table 2 presents the functional groups and peaks corresponding to each component of these two documents.

We notice a perfect overlapping of the peaks of the supports, but their intensities differ. The peak from 3287 cm^−1^ shows that there was registered valence vibrations ν for the -OH and -NH groups. They belong to amide A with a band range of 3400–3100 cm^−1^ and form the first component of the ν(NH) frequency in the Fermi resonance of amide II.

Amide B has the band between 3080 and 3070 cm^−1^ and is attributed to the second Fermi resonance, which is between the ν(NH) valence vibration and the end of amide II.

Amide I, with the band 1643–1630 cm^−1^ is attributed to the ν(C = O) frequency (valence vibrations of the peptide bonds).

Amide II, with the band 1550–1532 cm^−1^, is attributed to the ν(CN) valence vibration and the δ(NH) deformation vibration: respectively, the δ(CN) deformation vibrations.

Amide III, with the band 1240–1235 cm^−1^, the absorbance of which comes from δ(NH) and the δ(CH_2_) deformation vibration of glycine and the lateral chain of proline [17,18,19,20].

The peaks from 2917 cm^−1^ and 2849 cm^−1^ are specific to the valence bands of carboxylic groups.

The associated bands are representative in the IR spectral range of proteins (deformation vibrations), particularly the amides A_I_ and A_II_.

For assessing the rate of denaturation of collagen, we calculated the difference of positioning between the two amides and the ratio of their intensities:

P1: I(AI)/I(AII) = I(A1633)/I(A1540) = 0.136/0.147 = 0.93

P2: I(AI)/I(AII) = I(A1631)/I(A1542) = 0.094/0.095 = 0.99.

The value of the ratio of intensities of the two amides, which is approximately equal to 1, demonstrates that the parchment was slightly degraded by hydrolysis (in the case of the investigated micro-zones).

In general, this ratio of intensities of the bands of amides A_I_ and A_II_ is the indicator of the degree of breaking of the peptide bonds [21].

P1: Δν = ν(AI) − ν(AII) = 1633 − 1540 = 93 cm^−1^

P2: Δν = ν(AI) − ν(AII) = 1631 − 1542 = 89 cm^−1.^

The difference between the positions of the two amides, with the value of ≈93 cm^−1^, respectively 89 cm^−1^, demonstrates that an even more advanced form of degradation of the collagen fibers has occurred in the form of gelatinization or cornification, which is easily seen in the SEM microphotographs. These phenomena occur in the case of a prolonged hydration and repeated desiccations at high temperatures. In such cases, a thermal disorganization occurs (caused by the thermal activation energy, which imparts sufficient energy to the water molecules to compete with the hydrogen and the van der Waals bonds, which maintain the triple-helix configuration/helicoid structure), alongside the breaking of certain peptide links or the partial loss of the constitution water of the hydroxyl groups, or those between abutting amine and carboxyl groups.

In terms of degradation dynamics, the process of denaturation of collagen can be caused by inter- or intra-molecular changes at the level of the hydrogen bonds, as well as at the level of the constitutive amino acids, which leads to the destabilization of the ordered structure [22,23].

The same conclusion follows from the significant change in the FTIR spectra, which consists of the shifting of the peaks of amide II from 1550 to 1539 cm^−1^. The shift toward smaller numbers is due to the irreversible transformation of the native structure (triple helix) of the collagen into a disorganized structure (gelatinization) or a monolithic structure (cornification).

Moreover, the damage of the integrity of the triple helix of collagen is indicated by the ratio of the intensities of the bands from 1434 cm^−1^ (for P1) and 1415 cm^−1^ (for P2) and of amide III, which is

P1: I(A_1434_)/I(A_1236_) = 0.144/0.100 = 1.44

P2: I(A_1415_)/I(A_1235_) = 0.117/0.072 = 1.63.

The value is greater than 1, which demonstrates that the structure of the collagen was modified by cornification compared to the initial one. It is known that a collagen with an intact conformation of the triple helix (the integrity of the collage is unaffected) has a ratio between 1.0 and 0.5 [24,25].

Regarding the composition of the carbonate ion, peaks with values of 1435 cm^−1^ and 874 cm^−1^, which are characteristic to the absorption bands of calcium carbonate (CaCO_3_), confirm its presence in these two documents under scrutiny and originate from the treatments used during the technological process. The collagenic support S1 has all the intensities of the absorption bands smaller than the collagenic support S2, meaning that the first parchment is more affected than the second.

The deformation band found at 1079 cm^−1^ is attributed to the oxidation of the lateral chains of amino acids as well as to methionine, while the band at 1030 cm^−1^ can be attributed to δ(CN), which is found in amino acids such as glycine, proline, and hydroxyproline.

Another deformation band found at 712 cm^−1^ can be attributed to the O-C-O grouping from the CO_3_^2−^ ion, which is the allotropic form vaterite [26].

The ATR-FTIR spectra of the inks used for writing and decorating the two documents have similar peaks as the supports, with certain lower signals or shifts caused by diffusion of the ink into the support (Figure 12 and Table 2).

For the red pigment, peaks were identified at 1607, 1027, 1017, and 800 cm^−1^, which are attributed to cinnabar.

For green, the peaks are at 2165, 1411, 1319, 1034, 1027, and 828 cm^−1^, which are attributed to malachite.

For identifying the charcoal black in the writing and miniature ink, we used the peaks specific to tar remains from 1014 cm^−1^, where the most intense bands were registered. For the presence of calcium carbonate in the black ink, which can originate from the overlapping with the band of the calcium carbonate from the collagenic support, we identified the peaks at 1412 cm^−1^ and 875 cm^−1^.

In the case of golden inks used for writing and decorating the two documents on collagenic support, given that gold is a chemical element that does not absorb in IR, the majority of the peaks from the two spectra presented in Figure 12a,b, respectively, have lower absorbance than the other pigments, which is explained by the shielding of the support by the golden film.

## 4. Conclusions

Regarding the results from the technical analyses, we determined the chemical nature and the state of conservation of the support and inks.

Optical microscopy provided information on the color, porosity, roughness, granulation, and morphology of the microstructural components. Therefore, the analysis of the granular configuration, which was determined from the arrangement of the hair follicles, showed that these two documents were made of calf leather. With respect to the state of conservation of the inks used for writing and decorating these two documents, inks were applied onto the collagen support (first the black ink, then the red one). The degradations (reticular fissures/cracks, detachments of writing material, and the appearance of micro-lacunae) were caused by the fragilization in time of the binder as a result of the physical parameters of the climate, which strayed from the optimal values for storage, and of the different hydrophily of the constitutive materials.

The SEM analysis for the support and inks confirms the conclusion drawn from the OM analysis. By magnifying the areas analyzed, it was possible to highlight the microparticles of calcium carbonate and inorganic salt crystallites left as residues of the processing of the skins from which the parchment was produced.

The EDX analysis assessed the elemental composition of the constitutive materials of these two documents and provided information concerning the technologies employed for producing the supports. Therefore, S1 has a higher quantity of calcium, which is approximately five times higher than S2, meaning that the manufacturing technologies were different: for S1, the process involved the treatment with calcium hydroxide (slake lime), while for S2, the process employed either calcium hydroxide and sodium sulfite, for increasing the speed of removal of the other skin sub-products (epidermis and hypodermis, hair/wool, glandes, etc.).

By corroborating the data from the EDX and the ATR-FTIR analyses, we identified the natural pigments used for producing inks with which these documents were written and decorated: the natural pigment malachite (basic copper carbonate [2CuCO_3_Cu(OH)_2_]) for the green pigment; cinnabar for the red pigment; gold for the golden pigments; the binder of organic origin (gum arabic/xantan and slaked lime as mineral binder); carbon for the writing ink of S1; and in the case of S2, only a small quantity of carbon was added to the ferogallic ink.

The ATR-FTIR spectroscopy provided information regarding chemical changes caused by oxidation, hydrolysis, and denaturation processes of the collagen, which is the base material of the parchment, as well as the chemical changes of inks (particularly the binders). Moreover, it has been found that degradation was enhanced by the denaturation and gelatinization of the collagen, which led to its monolithization by compacting fibers, which present a lattice of three-dimensional fissures and micro-crevices resulting from fracturing during roll-up. These supports also registered dehydrations with the loss of the hygroscopic water, leading to contractions assisted by the elimination of the hydrogen bonds from the structure of the collagen’s polypeptide chains.

## Figures and Tables

**Table 1:** The elemental composition in gravimetric percentages for the samples collected from the two documents under scrutiny and indexed according to the area and type of material.

Sample	Elemental Composition (Gravimetric Percentages)														
	Na	K	Mg	Ca	Fe	Cu	Au	Hg	Al	C	Si	P	O	S	Cl
**S1-SD_1_**	0.807	1.065	0.254	3.779	-	-	-	-	0.169	40.733	0.460	-	51.498	0.543	0.691
**S1-SD_2_**	1.261	1.772	0.508	7.080	-	-	-	-	0.431	32.634	1.011	-	53.553	0.901	0.848
**S1-CN**	0.524	1.526	0.065	5.159	-	-	-	-	0.129	35.745	0.491	-	56.084	0.278	-
**S1-PR**	1.505	0.756	0.799	2.974	-	-	-	8.235	0.627	36.826	0.587	-	46.075	1.256	0.358
**S1-PA**	-	-	-	-	-	-	70.690	-	-	15.609	-	-	13.702	-	-
**S1-PV**	1.477	1.853	1.966	4.582	0.655	7.846	-	-	1.372	23.151	3.569	0.620	51.066	1.195	0.648
**S2-S**	1.171	1.032	0.213	1.295	-	-	-	-	0.359	41.772	0.377	0.097	52.154	0.635	0.895
**S2-CN**	0.292	1.622	0.055	8.391	5.842	-	-	-	0.109	26.024	0.768	0.152	53.781	2.702	0.262
**S2-CA**	-	-	-	2.135	-	-	52.851	-	-	20.121	-	-	24.893	-	-

**Table 2:** Representative peaks and spectral bands of the ions identified in the samples analyzed by ATR-FTIR.

Type Ion	Theoretical Spectral Bands (cm^−1^)	Peak Present in Samples (cm^−1^)	Samples Analyzed
Amide A	3400–3100	3287	S1-PR
		3277	S1-CN
		3304	S1-PA
		3281	S1-PV
		3284	S2-CN
Amide I	1643–1631	1642	S1-PR
		1633	S1-PA
		1630	S1-PV
		1630	S2-CN
Amide II	1550–1532	1540	S1-PR
		1545	S1-CN
		1540	S1-PA
		1546	S1-PV
Amide III	1240–1232	1238	S1-PR
		1233	S1-PA
		1234	S1-PV
Aluminosilicates	1175–860	1027	S1-PR
		1024	S1-CN
		1029	S1-PA
		1027	S1-PV
		1023	S2-CA
		1027	S2-CN
Sulfate and sulfide	1035–960	1017	S1-PR
		1014	S1-CN
Carbonate	890–800; 1100–1040; 1530–1320	800, 873; 1416; 1320	S1-PR
		874; 1414; 1321	S1-CN
		874; 1414	S1-PA
		828; 1411; 1319	S1-PV
		875; 1413	S2-CA
		875; 1412; 1322	S2-CN
Phosphate	920–830; 1900–1600; 2500–2150; 2900–2750	1600; 2157	S2-CN
		1607	S1-PR
		2165	S1-PV
Chloride	1050–900	1034	S1-PV
